# Health and equity implications of individual adaptation to air pollution in a changing climate

**DOI:** 10.1073/pnas.2215685121

**Published:** 2024-01-16

**Authors:** Matt S. Sparks, Isaiah Farahbakhsh, Madhur Anand, Chris T. Bauch, Kathryn C. Conlon, James D. East, Tianyuan Li, Megan Lickley, Fernando Garcia-Menendez, Erwan Monier, Rebecca K. Saari

**Affiliations:** ^a^Department of Civil and Environmental Engineering, University of Waterloo, Waterloo, ON N2L 3G1, Canada; ^b^School of Environmental Sciences, University of Guelph, Waterloo, ON N1G 2W1, Canada; ^c^Department of Applied Mathematics, University of Waterloo, Waterloo, ON N2L 3G, Canada; ^d^School of Medicine, Department of Public Health Sciences, University of California, Davis, CA 95616; ^e^School of Veterinary Medicine, Department of Medicine and Epidemiology, University of California, Davis, CA 95616; ^f^Department of Civil, Construction, and Environmental Engineering, North Carolina State University, Raleigh, NC 27695; ^g^Department of Earth, Atmospheric, and Planetary Sciences, Massachusetts Institute of Technology, Cambridge, MA 02139N; ^h^Department of Land, Air and Water Resources, University of California, Davis, CA 95616

**Keywords:** air pollution adaptation, health effects of climate change mitigation, modeling for sustainability, place-based approach, multi-sector dynamics

## Abstract

Future climate change can cause more days with poor air quality. This could trigger more alerts telling people to stay inside to protect themselves, with potential consequences for health and health equity. Here, we study the change in US air quality alerts over this century due to fine particulate matter (PM_2.5_), who they may affect, and how they may respond. We find air quality alerts increase by over 1 mo per year in the eastern United States by 2100 and quadruple on average. They predominantly affect areas with high Black populations and leakier homes, exacerbating existing inequalities and impacting those less able to adapt. Reducing emissions can offer significant annual health benefits ($5,400 per person) by mitigating the effect of climate change on air pollution and its associated risks of early death. Relying on people to adapt, instead, would require them to stay inside, with doors and windows closed, for an extra 142 d per year, at an average cost of $11,000 per person. It appears likelier, however, that people will achieve minimal protection without policy to increase adaptation rates. Boosting adaptation can offer net benefits, even alongside deep emission cuts. New adaptation policies could, for example: reduce adaptation costs; reduce infiltration and improve indoor air quality; increase awareness of alerts and adaptation; and provide measures for those working or living outdoors. Reducing emissions, conversely, lowers everyone’s need to adapt, and protects those who cannot adapt. Equitably protecting human health from air pollution under climate change requires both mitigation and adaptation.

## Significance

Air pollution is the leading environmental risk factor for early death. Alerts guide people to stay indoors when air quality is poor. Climate change can worsen air quality over this century. We show that this creates conditions for rising air quality alerts, disproportionately for racialized, unhoused, and poorly housed populations. Relying on people to protect themselves likely offers minimal benefits compared to reducing emissions; however, boosting adaptation can offer additional health benefits even under stringent climate policy. New policy could, for example, compensate people for moving indoors, and improve access to clean indoor air. We address active policy questions about how to equitably protect health under climate change, identifying levers for action against an increasing, unfair burden of air pollution.

Air pollution is the largest environmental threat leading to premature death (1) worldwide (2). It disproportionately affects vulnerable people in the United States (US), including in racialized (3, 4) and socio-economically disadvantaged (5) populations. On days with poor air quality, health authorities issue alerts guiding people to protect themselves by limiting their exposure to outdoor air. Alerts are triggered using an aggregate measure of air quality called the Air Quality Index (AQI) (6). Public responses to alerts vary with awareness, risk perception, social influences, and other factors (7, 8). Currently, 15 to 20% of Americans adapt to poor air quality, primarily by restricting their time outdoors (7).

Thanks to decades of improving air quality, alerts are rare for most Americans. Those improvements are at risk, however, due to climate change (9). While the literature on air quality alerts has examined their construction (10), communication (11–13), and compliance (7, 8), there is only one recent study of their health or economic value (focusing on the population over age 65 during 2014 to 2017) (14) and none on the effect of climate change and climate policy.

Climate change can worsen air quality (9, 15, 16). Climate policy can offer significant (17–23) and equity-improving (24) health benefits by improving air quality. Increasing adaptation could also reduce health risks. This has been shown empirically for the case of adapting to extreme temperature (25), but not yet examined for air pollution.

Here, we ask, “How many more air quality alerts may be triggered by climate change? Who will experience this rise in alerts, and how might it affect their health risks, and behavior to reduce those risks? How does that adaptation behavior affect health risks, and the associated benefits of climate policy? What can policymakers do to equitably address rising risks?”

To evaluate these questions, we estimate the rise in air quality alerts the in United States over this century due to climate change. We focus on the most harmful pollutant—outdoor fine particulate matter (PM_2.5_)—and its effects on the AQI and on premature death among adults (2).

We examine which populations are affected, their capacity to adapt to rising risks of premature death by moving indoors, and their per capita costs and benefits of adapting. We assess the extent to which people can compensate for their rising health risks by adapting. We examine the effectiveness of current practice and identify policy levers to promote adaptation. We compare the health benefits of adaptation—moving indoors to reduce exposures to outdoor air—to those of mitigation—reducing emissions to slow climate change and improve air quality.

To do this, we introduce estimates of the costs and benefits of adapting to outdoor air pollution by moving indoors. We quantify costs as foregone outdoor time, valued at the wage rate. We base benefits on the reduction in annual mean exposure to outdoor air pollution (PM_2.5_), achieved via daily decisions to stay indoors. Outdoor PM_2.5_ concentrations are from our prior work, covering the contiguous United States on a 1.9° × 2.5° grid (26). We account for exposure to outdoor PM_2.5_ while indoors via the infiltration factor (i.e., the fraction of outdoor PM_2.5_ that enters indoors and remains airborne) (27). The resulting reduction in exposure to outdoor PM_2.5_ lowers the risk of premature death from all causes, estimated with a concentration–response function relating outdoor PM_2.5_ to health risks (28) and valued based on willingness-to-pay to reduce that risk, following US regulatory impact analysis (29). We explore the potential for adaptation to protect health by considering a range of ways in which the population in each grid cell may decide to adapt, including complying with alerts issued at current or lowered PM_2.5_ concentration thresholds (“Threshold”), complying with alerts based on the behavior of others (“Social Learning”), adapting on days for which their benefits exceed their costs (“Rational actor”), and adapting to offset their rising health risks by as much as climate change mitigation (“Forced”). We perform distributional and sensitivity analyses to identify variables that influence the net benefits of adapting and to assess the uncertainty, variability, and equity implications of adaptation and adaptation policy.

We find that air quality alerts could increase steeply by the end of this century, especially in areas with high Black populations, higher incomes, and leakier homes. Moving indoors (adaptation) could theoretically be as protective of health as reducing emissions of outdoor air pollutants (mitigation), but that would likely come at a net cost and significant loss of outdoor time. Conversely, mitigation protects those who do not or cannot adapt. Even with mitigation, however, policy to promote more adaptation can offer net benefits. Such policy could, for example, lower adaptation costs or improve indoor air quality.

## Results

### Increasing Air Quality Alerts under Climate Change.

**Fig. 1.**
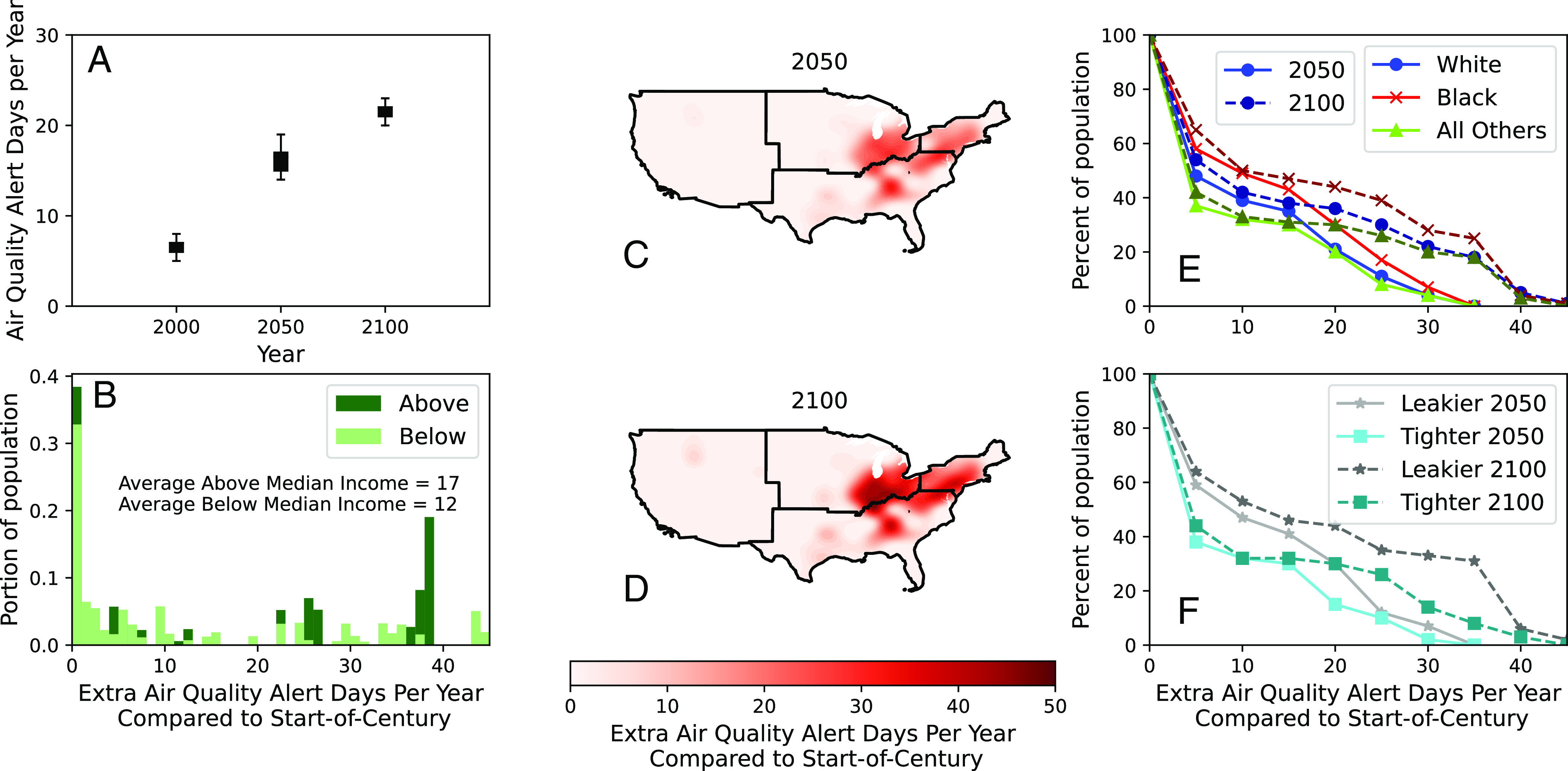
Air quality alert days per year (ADY) rise in the absence of emission reductions. All plots show air quality alerts (defined as outdoor fine particulate matter levels resulting in an Air Quality Index > 100) for the Reference (REF) climate change scenario. (*A*) National mean population weighted ADY for 2000, 2050, and 2100. Plots (*B*–*F*) show Extra ADY (EADY) compared to start-of-century (*B*) Histogram of EADY in 2100 for population above and below median income. (*C*/*D*) Spatial change in EADY in 2050 and 2100. (*E*) Cumulative density of EADY by race in 2050 and 2100. (*F*) Cumulative density of EADY by residential leakage rates above (“Leakier”) and below (“Tighter”) the national average. Leakage is defined as air changes per hour at a 50 Pa pressure difference (ACH50), indicating greater infiltration of outdoor air inside.

We find that, without reductions in emissions (of greenhouse gases or air pollutants), days with air quality alerts could quadruple on average by 2100 (Fig. 1). The rise is steepest over the eastern United States—increasing by 1 mo per year by 2100—coinciding with areas with high Black populations, higher incomes, and leakier homes.

We emphasize that, within those areas, this unequal rise can lead to disparities in individual capacity to adapt to rising alerts. Leakier buildings let more air pollution inside, rendering adaptation less effective. Housing with poor indoor air quality could even make adaptation harmful. Further, living in leakier housing is correlated with lower incomes (30), reducing adaptive capacity for this vulnerable group.

This finding suggests that rising alerts could potentially place an undue burden on marginalized groups based on race and socioeconomic status. Note that we project future air pollution spatially, and project total population, income, and baseline death rates as in our prior work (26) (details in *SI Appendix*), but rely on current demographics (specifying race but not Hispanic ethnicity), housing data, and time use. Those data derive from the US Census Bureau (31), National Renewable Energy Lab (27), and Bureau of Labor Statistics (32), respectively. The ultimate distribution of impacts will depend on future patterns of pollution, demographics, housing, health, and activity.

### Benefits of Adaptation and Mitigation, and Their Interaction.

This increasing burden of air pollution caused by climate change could be addressed through adaptation or mitigation. We compare these strategies in Table 1. This table shows per capita benefits of reducing premature mortality risks achieved by reducing exposure to PM_2.5_ of outdoor origin. That exposure reduction is achieved either via mitigation (here, reducing greenhouse gas emissions and thus reducing the worsening effect of climate change on air quality), adaptation (here, reducing exposures by moving indoors), or both.

**Table 1.** Benefits of individual adaptation and climate change mitigation at end-of-century associated with reduced mortality risk from outdoor air pollution (PM_2.5_)

Mitigation level	Adaptation level	Adaptation benefits ($) (95% CI)	Mitigation benefits ($) (95% CI)
Low	None	0	0
(REF)	Rational	$2,200 (200, 6,100)	0
	Forced	$5,400 (490, 15,000)	0
High (P3.7)	None	0	$5,100 (460, 14,000)
	Rational	$1,300 (120, 3,600)	$4,700 (430, 13,000)

National population weighted mean per capita benefits in 2020 USD for multiple levels of mitigation and adaptation. Values in parenthesis represent 95% CI in benefits related to health and economic uncertainty. REF: reference; temperature rise from pre-industrial by end-of-century is 6 °C. P3.7: meets Paris target; temperature rise from pre-industrial by end-of-century is 2 °C. Rational: all adults, acting with perfect information, maximize their net benefits of adapting. Forced: all adults, acting with perfect information, adapt until they achieve the same health protection as under mitigation (P3.7).

For climate change mitigation levels, we compare a reference (REF) case with a global carbon pricing policy meeting the 2 °C Paris Agreement target (P3.7). The reference case (REF) (also used in Fig. 1) has a mean global surface temperature rise at end-of-century (ΔT) compared to the preindustrial period (1850 to 1869) of 5.7 °C. The climate policy (P3.7) has a ΔT of 1.9 °C. These scenarios are from our previous work, using models of the global economy and Earth system (33) (details in *Materials and Methods* and *SI Appendix*).

Table 1 shows individual adaptation (by moving indoors) could offer benefits similar to—or even larger than—mitigation; however, those benefits of adaptation come with a significant burden. This is shown by comparing the benefits of “forced” adaptation to that of mitigation without additional adaptation: $5,400 ($490 to $15,000) and $5,100 ($460 to $14,000) population-weighted mean per-capita benefits, respectively. Forced adaptation requires the full adult population to fully compensate for its increased exposure, acting with perfect knowledge of its risks and benefits. Since we assume people only spend 1 h per day outside, on average, across all outdoor activities (details in *Materials and Methods*), it would take an additional 142 d per year, on average, to achieve the same reduction in exposure afforded by climate change mitigation policy.

Table 1 also shows that the greatest benefit is achieved with both mitigation and adaptation ($6,000 per person as the sum of benefits under P3.7). Their combination also tends to reduce the effectiveness of each strategy when used alone. Climate change mitigation policy (P3.7) tends to reduce the need for adaptation. Under climate policy, per capita benefits of adapting drop from a mean of $2,200 ($200 to $6,100) to $1,300 ($120 to $3,600). The remaining $1,300 benefit per person implies that, even under climate policy, there are gains to be made by boosting adaptation. Similarly, when estimating the benefits of mitigation, we find a small reduction in benefits due to adaptation. This interaction occurs because the adaptors staying inside are less exposed to outdoor air, so receive less benefit from improving outdoor air quality. This effect reduces per capita benefits of mitigation, however slightly, from $5,400 ($490 to $15,000) to $4,700 ($430 to $13,000).

### Adaptation Responses to Air Quality Alerts under Climate Change.

If air quality worsens, the burden of air pollution will rise, either in terms of higher health risks, or higher adaptation costs. In Table 2, we examine a range of possible adaptation behaviors to explore how protective individual adaptation might be. We evaluate our reference climate change scenario at end-of-century. We present uncertainty in the health and economic benefits of adapting derived from our prior work using 5,000 Monte Carlo simulations in the US Environmental Protection Agency (EPA) health impact software, the environmental Benefits Mapping and Analysis Program Community Edition (BenMAP-CE) (26, 34). Uncertainty in costs is addressed later.

**Table 2.** Potential future net benefits of adapting to air pollution

Threshold for air quality advisory	Compliance level	Adaptation cost ($)	Adaptation benefits ($) (95% CI)	Adaptation net benefits ($)
N/A		0	0	0
Current (AQI > 150)	Current	$110	$210 (20, 580)	$100
Lowered (AQI > 100)	Current	$340	$440 (40, 1,200)	$100
Current (AQI > 150)	Socially-influenced compliance	$340	$700 (60, 2,000)	$360
Lowered (AQI > 100)	Socially-influenced compliance	$1,200	$1,700 (150, 4,700)	$480
Current (AQI > 150)	Full compliance by adult population	$550	$1,000 (90, 2,900)	$500
Lowered (AQI > 100)	Full compliance by adult population	$1,700	$2,200 (200, 6,200)	$530
Rational behavior	Maximum net benefits of adaptation	$1,400	$2,200 (200, 6,100)	$780
Forced	Forcing adaptation to same risk reduction as mitigation	$11,000	$5,400 (490, 15,000)	−$5,900

**Fig. 2.**
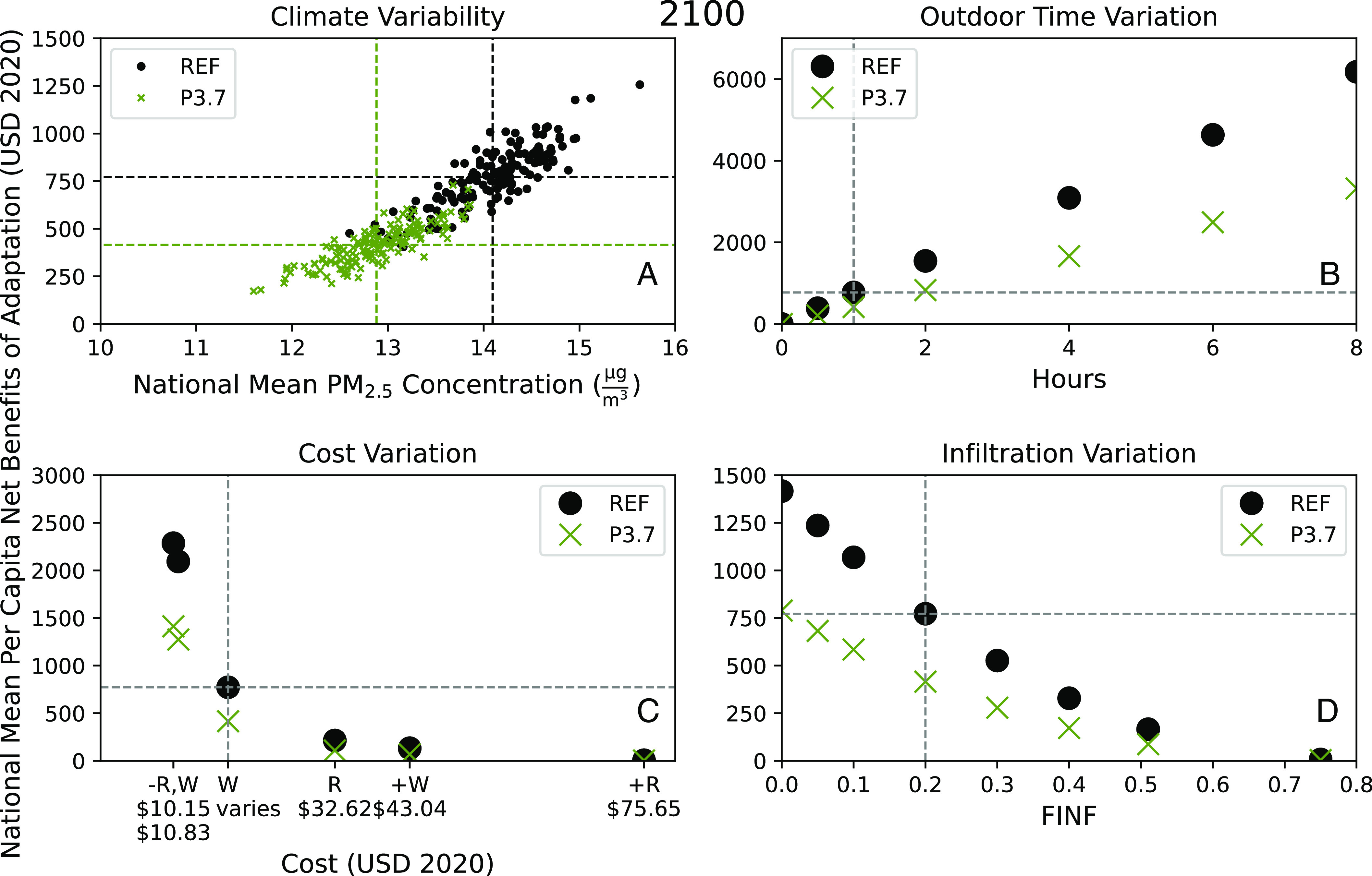
Variation of individual adaptation net benefits per capita under different levels of climate change mitigation in 2100 (in 2020 USD). (*A*) Scatter plot comparing national mean per capita net benefits of adaptation to national mean PM_2.5_ concentrations under REF and P3.7. Horizontal dashed lines represent population-weighted mean per capita net benefits over an ensemble of 150 annual simulations representing end-of-century. Vertical dashed lines represent the corresponding mean population-weighted PM_2.5_ concentrations. For (*B*–*D*), the vertical dashed lines represent the national mean of the dependent variable, and horizontal dashed lines show mean net benefits under REF. (*B*) Variation with amount of daily outdoor time given up. (*C*) Variation with cost per hour of adaptation. R represents costs derived from recreation values; W represents costs derived from wage values. − and + represent the minimum and maximum values applied nationally, respectively. R is the mean of all recreation values reported by Loomis (35). W applies the wage for each grid cell aggregated from that grid cell’s corresponding county-level data (36). (*D*) Variation with the amount of outdoor pollution reaching indoors [as Infiltration Factor (FINF)].

National population weighted per capita net benefits (2020 USD) in the adult population for various levels of adaptation to air pollution in the absence of climate change mitigation by end-of-century. Adaptation reflects the current dominant approach, which is to limit time outdoors. Adaptation levels are varied by changing the threshold for air quality advisories, compliance levels, and decision approaches. For benefits, values in parenthesis represent the 95% CI due to uncertainty in health and valuation. Note that uncertainty in costs is not included here, but is explored in Fig. 2.

We first consider that there may be no additional adaptation, offering no protection at no cost—a common assumption in most studies of the future health impacts of air pollution. Next, we evaluate current practice (“Threshold”), which issues alerts to the general population when the AQI exceeds 150, and to the sensitive population when AQI exceeds 100. Here, we estimate the AQI using only outdoor PM_2.5_ and its respective thresholds of 55.4 μg/m^3^ and 35.4 μg/m^3^. We use the current number of Americans who adapt to air pollution, estimated to be near 20%(7, 7) (7) The top 20% most exposed Americans experience five alert days per year (*SI Appendix*, Fig. S2); we estimate that this will grow by more than a month by 2100 (Fig. 1*E*). If current adaptors comply with rising alerts, this amounts to national mean benefits of $210 ($20 to $580) per person per year by 2100. Removing costs, mean net benefits are $100 per person per year by 2100. Lowering the threshold for alerts from an AQI of 150 to 100—the value used for populations that are particularly sensitive to air pollution—has little effect on mean net benefits of adaptation.

It also is possible that more people will begin to comply with alerts. We examine the potential rise in adaptation four ways: 1) Social Learning allowing people to learn compliance, 2) full compliance of the adult population to all air quality alerts (meaning all adults stay indoors on alert days), 3) all adults maximize their net benefits (benefits–costs) of adapting, using perfect information (Rational actor), 4) adults are forced to adapt until they reduce their exposures by the same amount as climate change mitigation (Forced). We present national population-weighted mean results, noting that the spatial distribution of adaptation net benefits is similar to the rise in alerts shown in Fig. 1*D* for Social Learning, Rational actor, and Threshold (full compliance).

We first assess the potential rise in adaptation due to social learning. We use a model (Social Learning) allowing people to switch between compliance and non-compliance, based on their perceived benefits of doing so (details in *Materials and Methods* and *SI Appendix*). Those perceived benefits vary with pollution, costs, and the behavior of others, allowing them to learn compliance (or non-compliance) from others, and adapt to changing environmental conditions and social norms. Those who comply will give up some fraction of their outdoor time during alerts.

We find this social learning process could lead to higher adaptation rates in the future, on average, tripling the mean net benefits of adapting over current practice. This suggests that, even absent new adaptation policy, social influence could affect adaptation rates.

Nonetheless, it appears likely that the net benefits of adaptation will remain small compared to those of mitigation (P3.7); in fact, forcing the benefits to match appears detrimental. This is seen by comparing the net benefits of full compliance, rational behavior, and forced adaptation. Each approach increases the average benefits of adaptation, with full compliance offering similar benefits to rational behavior. Forcing the adult population to reduce its exposure to outdoor PM_2.5_ to the same level as climate policy (P3.7) offers the most benefits, by requiring people to stay indoors on many days without alerts. Though this achieves per capita benefits $5,400 ($490 to $15,000) higher than our mitigation scenario ($5,100), the costs are $11,000 per person, meaning this approach is detrimental, incurring an average net loss of $5,900 per person.

### Policy Levers to Promote Adaptation.

Policy to promote adaptation could potentially offer net benefits. Robust and equitable policy, however, should consider the uncertainty, individual variability, and distribution of net benefits. To support this, we present a sensitivity analysis of factors affecting the maximum achievable net benefits (using Rational actor behavior) (see Fig. 2).

Adaptation offers higher net benefits to those who live in polluted areas, who work and live in high-quality buildings, and who regularly spend time outdoors but place a low value on this time. This result is shown by the variation of net benefits of adaptation in Fig. 2 *A*–*D*: increasing with a) concentration and b) time outdoors, decreasing with d) adaptation costs, and e) infiltration. We present variation in adaptation costs as the value of outdoor time based on variation in wages and from economic studies of the value of outdoor recreation (35).

Additional adaptation cannot benefit those who never go outdoors, have leaky homes (F_INF_ > 0.5), or who highly value their outdoor time. A population with these characteristics may highly prefer mitigation to adaptation.

Mitigation reduces the need to adapt for all and protects even those who cannot adapt. It also decreases variability in the benefits of adapting. This is seen by comparing the green (mitigation) to black (reference) cases for all subplots of Fig. 2. Mitigation reduces annual mean PM_2.5_ concentrations by 1.2 µg/m^3^, lowering the benefits of adapting. We saw this in Table 1, too, but Fig. 2*A* shows that this holds well despite the variability in future annual mean concentrations. This means, too, that the rate of change of net benefits is smaller across all other variables in Fig. 2.

To boost adaptation, policy can reduce adaptation costs, improve building quality, increase awareness, and should consider options for outdoor workers and people experiencing homelessness. We mention increasing awareness based on our social learning model; we showed that social learning could increase adaptation in Table 2. From Fig. 2, we see the greatest gain in adaptation benefits is possible by minimizing adaptation costs, then reducing infiltration. This suggests policy levers including compensating people for moving indoors and improving access to clean air. For most Americans, who live and work indoors, this could be achieved by improving indoor air quality. Fig. 2 also shows that the highest possible gains from adaptation are for those who spend at least 8 h outdoors, a group consisting mainly of outdoor workers, and people experiencing homelessness, each of whom require specific interventions.

## Discussion and Conclusions

Climate change is known to raise risks to human health, increasing the need for adaptation to protect health (37). We examine the potential effect of climate change on air quality alerts and adaptation by moving indoors. We find that unabated climate change triggers more air quality alerts guiding people to protect themselves by moving indoors, quadrupling on average by the end of this century.

Those rising alerts could exacerbate existing racial inequalities and disparities in adaptive capacity. For example, we find alerts rise by 1 mo per year in the Eastern United States, in areas that currently have higher Black populations (31), and leakier homes that let more outdoor air pollution inside (27).

Boosting adaptation beyond current levels could offer net benefits, especially under worsening air quality, but even if Paris Climate Agreement targets are met. That policy could involve compensating people for moving indoors, improving building quality, and increasing awareness. Awareness could be raised through multimedia campaigns and communication channels targeted to relevant demographics (11–13), including, for example, integrating air quality messaging into patient charts (13).

For some, additional adaptation may be impossible, or even harmful, without appropriate intervention. It cannot benefit those who spend their time in leaky buildings (F_INF_ > 0.5) or who cannot give up their outside time, either because they do not go outside, because they value it highly, or because it is required for their work [true for approximately 4% of civilian workers (38)], or because they are experiencing homelessness [approximately 0.18% of the US population (39)].

Thus, in order to effectively and equitably protect human health from air pollution, policy for adaptation should be considered alongside mitigation. Mitigating climate change can the increased risk from worsening air quality and protects those who cannot adapt. Adaptation alone could pose an unrealistic substitute for improving air quality, in our case, requiring an additional 142 d per year of adaptation beyond current levels by 2100. At the same time, it remains highly uncertain how much climate change mitigation will be achieved, and how this will ultimately affect outdoor PM_2.5_, meaning that enhanced adaptation may be needed (40).

Combinations of mitigation and adaptation should be evaluated and leveraged. We find the highest total benefits from both mitigation and adaptation, though the full distribution of costs and benefits must be assessed. We also find that their interaction reduces the effectiveness of each strategy alone. There remain other important feedbacks between air quality, climate change, and adaptation to unravel. Prior work has shown various effects of staying indoors on air pollution, including potential positive feedbacks of reduced traffic (41), negative feedbacks of increased heating and cooling demand (42), and feedbacks between climate change, cooling demand, and air pollution (43). Some of these feedbacks can be leveraged to offer multiple benefits. For example, improving building quality to reduce infiltration and improve indoor air quality not only increases the benefits of adapting, it also reduces exposure to outdoor air pollution during all time indoors. It could also reduce energy demand for heating and cooling, with positive effects for climate change and outdoor air quality.

Our work is in line with prior estimates of the benefits of adapting to air pollution, given study differences. Buonocore et al. (14) find per capita benefits to be no higher than $14 per person per hour in the population aged 65 and over in 2014 to 2017. Here, we find an average benefit of $31 per person per hour for adults aged 30 and over at the start-of-century across the United States. The most important reason for this difference is that we account for the fact that people spend most of their time indoors, where exposure to PM_2.5_ of outdoor origin is lower, therefore, exchanging 1 h outdoors for indoor time offers a greater reduction in overall exposure to PM_2.5_ of outdoor origin. When we reproduce (14)’s findings with our estimate of infiltration, we find up to a 1.5-fold (or 50%) increase in benefits. We also do not include morbidity or ozone, but these reflect less than 10% of our benefits (*SI Appendix*, Table S2).

In order to inform policy to protect health from outdoor air, we focus on PM_2.5_ of outdoor origin. We represent the main risk-cost trade-offs in air quality policy analysis (PM_2.5_-associated premature death), existing adaptation guidance (via air quality alerts), and the current dominant adaptation mode (limiting time outdoors). We estimate the effects of climate change on air pollution, including anthropogenic and biogenic sources, but do not account for wildfires, which could also exacerbate air pollution under climate change (44), enhancing benefits of adaptation beyond our estimates. Focusing on outdoor PM_2.5_ ignores PM_2.5_ that originates indoors. For adaptation to be protective, individuals must be able to move indoors to relatively clean environments. Here, we conservatively estimate 20% infiltration of outdoor PM_2.5_ indoors, while new US homes may have infiltration factors as low as 1 to 2% (45). Low-cost PM_2.5_ filtration is available to the two-thirds of US households using central air-conditioning (46). However, indoor sources of PM_2.5_, such as tobacco smoking, cooking, and the use of consumer products, may substantially increase indoor PM_2.5_ concentrations (47–49) and could represent comparable exposures as outdoor sources (50, 51). Further work is needed to understand how increased adaptation to outdoor pollution could affect total PM_2.5_ exposure and associated risks to human health. Similarly, significant shifts from outdoor to indoor activities could have multiple impacts to well-being not considered here, particularly if physical activity were affected. Current guidance from the American Thoracic Society is not to reduce physical activity in response to poor air quality, but to shift it to locations and times when pollution is lower (52). Future work should consider the broader health implications of adaptation.

Our work contributes to challenges in nature–society systems modeling on cross-scale effects (e.g., time scales, scales of impact and action), representing social responses, and addressing uncertainty (53–55). We build on recent examples focusing on climate change, behavior, and social dynamics and the effects on mortality and policy outcomes (e.g., refs. 25 and 56–58). We represent several approaches to modeling adaptation decisions to assess their effect on nature–society interactions (59). This includes a simple model of social learning based on replicator dynamics, whose theory, behaviors, and applications in nature–society models we recently reviewed (60). That model fits well to historical, nationally representative surveys of adaptation behavior (details in *SI Appendix*). However, other theories of behavior could also explain these data, beyond those explored here. Behavior could also evolve over time, e.g., due to changing risks, risk perception, activities, or valuation. We present ranges of net benefits of adaptation across physical and behavioral characteristics to inform more detailed demographic analysis and projection. Representing human behavior in nature–society systems models remains a frontier capability with much future work ahead (61).

## Materials and Methods

### Air Quality Concentrations.

This work derives future air quality concentrations from previous work (9, 26, 62). That work employed the Massachusetts Institute of Technology (MIT) Integrated Global System Model (IGSM) framework (33). This framework links a global computable general equilibrium economic model [Economic Projection and Policy Analysis (EPPA)], an earth system model of intermediate complexity [MIT Earth System Model (MESM)], and an air quality model [the National Center for Atmospheric Research (NCAR) Community Atmosphere Model with Chemistry (CAM-Chem)] to generate air pollution concentrations for scenarios as in refs. 9, 26, and 62 and summarized in *SI Appendix*, Table S6. Modeled pollutant concentrations correspond well with measured values, with year 2000 national simulated annual population weighted PM_2.5_ concentration within 7% of the EPA’s reported national average (62).

### Air Quality Alerts.

Air quality alerts are typically issued when the Air Quality Index (AQI), as defined by the US EPA (6), exceeds a threshold. We study the current thresholds for the general (150) and sensitive (100) populations. In practice, AQI values are calculated separately for multiple pollutants, and the highest value is reported as the AQI. For this study, we only consider PM_2.5_ concentrations, with AQI thresholds of 100 and 150 corresponding to 24-h mean PM_2.5_ concentrations of 35.4 μg/m^3^ and 55.4 μg/m^3^, respectively.

### Adaptation Net Benefits.

We model adaptation behavior in the adult (age 25 to 99) contiguous US population. We represent avoidance—moving indoors to reduce exposure—the current dominant mode of adaptation (7). Daily 24-mean PM_2.5_ concentrations are analyzed at the air quality model grid scale (1.9° × 2.5°) for each annual simulation. Annual simulations represent three sets of three decades, including 1981 to 2010, 2036 to 2065, and 2086 to 2115, for each of five sets of initial climate conditions. We average results to estimate conditions at start, mid, and end-of-century under a reference (REF) and climate policy scenario (P3.7).

Population and demographic data are aggregated to the air quality model grid. Populations make daily adaptation decisions. The value of adapting on a given day *d* is described its net benefits per Eq. **1**.

[1] $${\mathrm{NB}}_{d}=B_{d}-C_{d},$$

where ${\text{NB}}_{d}$   is the net benefits of adapting on day *d*, calculated as $B_{d}$   , the benefits of adapting on day *d*, minus $C_{d}$ , the cost of adapting on day *d*.

### Adaptation Benefits.

Adaptation lowers daily PM_2.5_ exposure, which also lowers annual average exposure, reducing the incidence rate of premature mortality. The equivalent daily benefit of this risk reduction is shown in Eq. **2**.

[2] $$B_{d}=\text{VSL}\times \Delta I_{d},$$

where VSL represents the Value of a Statistical Life, and $\Delta I_{d}$ is the change in incidence rate of premature mortality achieved by adapting on day *d*.

VSL is a measure of willingness-to-pay to reduce the risk of premature mortality. We use the most recent value provided by the US EPA, based on a review of 26 studies, 7.9 million USD (2008) (29).

We calculate the change in incidence of premature mortality from all causes from adapting on day *d* with Eq. **3**.

[3] $$\Delta I_{d}=Y_{0}\times \frac{\text{RR}-1}{\text{RR}}\times \frac{{\text{ΔPM}}_{2.5,a_{d}}}{10\text{μg}/m^{3}},$$

where $Y_{0}$ is the population’s baseline mortality rate, RR is the relative risk of increased premature all-cause mortality per $10\text{μg}/m^{3}$ increase in outdoor PM_2.5_ concentration, and ${\text{ΔPM}}_{2.5,d}$ is the difference in equivalent annual outdoor PM_2.5_ concentration after adaptation on day *d*.

As in our prior work (26), $Y_{0}$ varies by grid cell and year (2000, 2050, and 2100) using start-of-century values from the environmental Benefits Mapping and Analysis Program community edition (BenMAP-CE), and projected using International Futures, with details in *SI Appendix*, Table S9. RR is 1.14 (28).

We calculate ${\text{ΔPM}}_{2.5,a_{d}}$ with Eq. **4**.

[4] $${\text{ΔPM}}_{2.5,a_{d}}={\text{PM}}_{2.5,a}-{\text{PM}}_{2.5,a_{d}}^{’},$$

where ${\text{PM}}_{2.5,a}$ is the annual mean outdoor concentration of PM_2.5_ with no adaptation and ${\text{PM}}_{2.5,a_{d}}^{’}$ is the annual mean outdoor equivalent concentration of PM_2.5_ after adaptation on day *d*.

We calculate ${\text{PM}}_{2.5,a}$ with Eq. **5**.

[5] $${\text{PM}}_{2.5,a}=\frac{\sum_{n=1}^{365}{\text{PM}}_{2.5,n}}{365},$$

where ${\text{PM}}_{2.5,n}$ is the 24-h mean outdoor concentration of PM_2.5_ on each nonadaptation day *n* of the 365 d in year *a*.

We calculate ${\text{PM}}_{2.5,a_{d}}^{'}$ with Eq. **6**.

[6] $${\text{PM}}_{2.5,a_{d}}^{'}=\frac{\sum_{n=1}^{364}{\text{PM}}_{2.5,n}+\psi \times {\text{PM}}_{2.5,d}}{365},$$

where ${\text{PM}}_{2.5,n}$   is the 24-h mean outdoor PM_2.5_ concentration on each non-adaptation day *n*, and ${\text{PM}}_{2.5,d}$ is the 24-h mean outdoor PM_2.5_ concentration on the adaptation day *d*. ψ is the ratio of exposure to outdoor PM_2.5_ when adapting (i.e., spending all day indoors) compared to not adapting (i.e., spending some of the day outdoors) given by Eq. **7**.

[7] $$\psi =\frac{24\times F_{\text{INF}}}{T_{\text{out}}+\left(24-T_{\text{out}}\right)\times F_{\text{INF}}},$$

where $T_{\text{out}}$ is the average daily time normally spent outdoors in hours, and $F_{\text{INF}}$ is the infiltration factor.

ψ thus quantifies the exposure reduction achieved by adapting, while accounting for exposure to outdoor PM_2.5_ while indoors. We use a $T_{\text{out}}$ of 1 h based on the American Time Use Survey (ATUS) (32) and to ease comparison with previous studies (14). We therefore assume that non-adaptors spend 23 h indoors. We use an $F_{\text{INF}}$ value of 0.2 for this study based on surveys of the US housing stock in refs. 63 and 64.

### Adaptation Costs.

We calculate adaptation costs, $C_{d}$ , with Eq. **8**.

[8] $$C_{d}=\phi \times \xi \times T_{\text{adapt}},$$

where ϕ represents the hourly adaptation cost, ξ is the portion of outdoor time given up for adaptation, and $T_{\text{out}}$ is the time spent outdoors on a non-adaptation day. For all strategies, the default hourly adaptation cost for a grid cell is that grid cell’s mean hourly wage, aggregated from county level US census data.

### Adaptation Decision-Making Strategies.

We use these benefits and costs to study the effectiveness of short-term avoidance adaptation in reducing outdoor PM_2.5_-related premature mortality risk. We estimate adaptation behavior with four models: Rational actor, Threshold, Forced, and Social Learning. These strategies determine the number of days of adaptation (d) and non-adaptation (n).

#### Rational actor.

Our Rational actor model represents an adult population acting with perfect information to adapt on any day with positive net benefits. We model this for each grid cell by sorting days of the year by decreasing marginal benefits of adapting that day. This uses Eq. **2** for all days in a year sorted from most to least polluted. The marginal costs of adapting an additional day are always given by Eq. **8** with ξ=1 . We then calculate ${\mathrm{NB}}_{d}$ (with Eq. **1**). All days with ${\text{NB}}_{d}\geq 0$ are adaptation days; once ${\text{NB}}_{d}<0$ adaptation stops for all less polluted days, with ${\text{NB}}_{d}$ , $B_{d}$ , and $C_{d}$ set to zero.

#### Threshold.

Our Threshold model is based on current adaptation guidance, during which alerts are triggered by the AQI. We use thresholds of AQI > 100 and AQI > 150. This population of compliant adults adapts if the AQI in their grid cell exceeds the threshold, accruing costs (per Eq. **8** with ξ=1 ) and benefits (per Eq. **2**). We compare compliance levels at current rates (20%) to that of a theoretically fully compliant population.

#### Forced.

Our Forced model considers the reference climate change scenario (REF) and requires the full adult population to reduce their exposures to those in our climate change mitigation scenario (P3.7). This population acts like our Rational actors who are willing—irrationally—to continue adapting beyond the point that benefits exceed costs, stopping only when their resulting annual mean PM_2.5_ concentrations (per Eq. **6**) in their grid reach those of the annual mean outdoor concentrations of P3.7 (per Eq. **5**).

#### Social learning.

In the Social learning model, populations are partitioned into adapters and non-adapters. At each model time-step, adaptors and non-adaptors decide whether to switch groups based on the relative utility of their two strategies. Like the Threshold model, those in the adapter group adapt when the PM_2.5_-based AQI in their grid cell is greater than the study threshold (either 100 or 150). ${\text{NB}}_{a}$ is calculated by summing ${\text{NB}}_{d}$ , $B_{d}$ , and $C_{d}$ for the proportion of the population that adapted.

Over time, the proportion of adapters is governed by the discrete social learning model, advancing by time-steps of one quarter, or 3-mo period, to account for seasonal variation in pollution. This is calculated via Eq. **9**.

[9] $$x_{i,t+1}^{\sim }=x_{i,t}+\kappa_{r}\sigma_{i}x_{i,t}\left(1-x_{i,t}\right)\Delta U_{i,t},$$

where $x_{i,t}$   is the proportion of adapters in grid cell *i* at time *t*, $x_{i,t+1}$ is the proportion of adapters at the next time step, bound between 0 and 1, and $\Delta U_{i,t}$ is the difference in utility between adaptation and non-adaptation (in *SI Appendix*). $\kappa_{r}$ denotes the social learning rate in each US census region and $\sigma_{i}$ is the population of gridcell *i*.

Population is included because higher population areas lead to more diverse social networks for inhabitants which increases the spread of information (65). The proportion of adapters in a grid cell is bound between 0 (no adapters in the population) and 1 (the entire population adapts), where the tilde above *x* in Eq. **9** reflects this bounding.

We calculate $\Delta U_{i,t}$ in Eq. **10**.

[10] $$\Delta U_{i,t}=\frac{R_{i,t}-C_{i,t}}{R_{0}}+\delta_{r}\left(2\omega \left(x_{i,t}\right)-1\right),$$

where $C_{i}$   represents the cost associated with adaptation in grid cell *i*. $R_{i}$   is a function that represents the perceived risk of negative health effects due to air pollution in region *i*. An adapter believes that they mitigate their risk of these negative health effects by adapting, whereas a non-adapter receives the full amount of this perceived health risk. $\delta_{r}$   represents the weight of social norms in each US census region and $\omega (x_{i})$   is a function that determines the social norm that is predominant for grid cell *i*. $R_{0}$   normalizes the level of perceived utility from individual action to a similar magnitude as the influence of social norms. Cost has the same form as Eq. **8**, but summed over 4 mo, and with ξ fit empirically and varying by US census.

This model is fit to representative surveys of US adaptation (7), aggregated by US Census region, from 2014 to 2020 with adjusted R^2^ for the four regions ranging between 0.56 and 0.80. *SI Appendix* contains further explanation of the Social Learning Model development and performance.

## Supplementary Material

Appendix 01 (PDF)

## Data Availability

Anonymized code and data have been deposited in Github and Dataverse (https://github.com/mattsparks78/AQ-adaptation; https://doi.org/10.5683/SP3/U3XQJH). Previously published data were used for this work (9).

## References

[1] R. Burnett , Global estimates of mortality associated with long-term exposure to outdoor fine particulate matter. Proc. Natl. Acad. Sci. U.S.A. **115**, 9592–9597 (2018). 30181279 10.1073/pnas.1803222115 PMC6156628

[2] A. J. Cohen , Estimates and 25-year trends of the global burden of disease attributable to ambient air pollution: An analysis of data from the Global Burden of Diseases Study 2015. Lancet **389**, 1907–1918 (2017). 28408086 10.1016/S0140-6736(17)30505-6 PMC5439030

[3] C. W. Tessum , PM2.5 polluters disproportionately and systemically affect people of color in the United States. Sci. Adv. **7**, eabf4491 (2021). 33910895 10.1126/sciadv.abf4491 PMC11426197

[4] J. Liu , Disparities in air pollution exposure in the United States by race/ethnicity and income, 1990–2010. Environ. Health Perspect. **129**, 127005 (2021). 34908495 10.1289/EHP8584 PMC8672803

[5] N. Z. Muller, P. H. Matthews, V. Wiltshire-Gordon, The distribution of income is worse than you think: Including pollution impacts into measures of income inequality. PLoS One **13**, e0192461 (2018). 29561838 10.1371/journal.pone.0192461 PMC5862398

[6] United States Environmental Protetion Agency, Technical Assistance Document for the Reporting of Daily Air Quality – the Air Quality Index (AQI) (2018) (May 4, 2022).

[7] M. C. Mirabelli, S. Ebelt, S. A. Damon, Air quality index and air quality awareness among adults in the United States. Environ. Res. **183**, 109185 (2020). 32007750 10.1016/j.envres.2020.109185 PMC7182097

[8] K. Bickerstaff, Risk perception research: Socio-cultural perspectives on the public experience of air pollution. Environ. Int. **30**, 827–840 (2004). 15120202 10.1016/j.envint.2003.12.001

[9] F. Garcia-Menendez, R. K. Saari, E. Monier, N. E. Selin, U.S. Air quality and health benefits from avoided climate change under greenhouse gas mitigation. Environ. Sci. Technol. **49**, 7580–7588 (2015). 26053628 10.1021/acs.est.5b01324

[10] J. Yao, D. M. Stieb, E. Taylor, S. B. Henderson, Assessment of the Air Quality Health Index (AQHI) and four alternate AQHI-Plus amendments for wildfire seasons in British Columbia. Can. J. Public Health **111**, 96–106 (2019). 31286460 10.17269/s41997-019-00237-w PMC7046905

[11] G. Xu, X. Feng, Y. Li, J. Jia, Mediation effects of online public attention on the relationship between air pollution and precautionary behavior. J. Manage. Sci. Eng. **7**, 159–172 (2021).

[12] R. Riley , How do we effectively communicate air pollution to change public attitudes and behaviours? A review. Sustain. Sci. **16**, 2027–2047 (2021).

[13] L. K. Tompkins, A. F. Pennington, K. D. Sircar, M. C. Mirabelli, Communication channels for receiving air quality alerts among adults in the United States. Prev. Med. Rep. **25**, 101677 (2022). 35127356 10.1016/j.pmedr.2021.101677 PMC8800048

[14] J. J. Buonocore, L. A. Robinson, J. K. Hammitt, L. O’Keeffe, Estimating the potential health benefits of air quality warnings. Risk Anal. **41**, 645–660 (2021). 33249613 10.1111/risa.13640

[15] A. M. Fiore , Characterizing changes in eastern U.S. pollution events in a warming world. J. Geophys. Res. Atmos. **127**, e2021JD035985 (2022).

[16] J. L. Schnell, M. J. Prather, Co-occurrence of extremes in surface ozone, particulate matter, and temperature over eastern North America. Proc. Natl. Acad. Sci. U.S.A. **114**, 2854–2859 (2017). 28242682 10.1073/pnas.1614453114 PMC5358352

[17] J. J. West , Co-benefits of mitigating global greenhouse gas emissions for future air quality and human health. Nat. Clim. Change **3**, 885–889 (2013). 10.1038/NCLIMATE2009 PMC4051351 24926321

[18] T. M. Thompson, S. Rausch, R. K. Saari, N. E. Selin, A systems approach to evaluating the air quality co-benefits of US carbon policies. Nat. Clim. Change **4**, 917–923 (2014).

[19] R. K. Saari, N. E. Selin, S. Rausch, T. M. Thompson, A self-consistent method to assess air quality co-benefits from U.S. climate policies. J. Air Waste Manage. Assoc. **65**, 74–89 (2015). 10.1080/10962247.2014.959139 25946960

[20] T. M. Thompson, S. Rausch, R. K. Saari, N. E. Selin, Air quality co-benefits of subnational carbon policies. J. Air Waste Manage. Assoc. **66**, 988–1002 (2016). 10.1080/10962247.2016.1192071 27216236

[21] M. Li , Air quality co-benefits of carbon pricing in China. Nat. Clim. Change **8**, 398–403 (2018).

[22] C. L. Gallagher, T. Holloway, Integrating air quality and public health benefits in U.S. decarbonization strategies. Front Public. Health **8**, 563358 (2020). 33330312 10.3389/fpubh.2020.563358 PMC7717953

[23] D. Shindell , Temporal and spatial distribution of health, labor, and crop benefits of climate change mitigation in the United States. Proc. Natl. Acad. Sci. U.S.A. **118**, e2104061118 (2021). 34725255 10.1073/pnas.2104061118 PMC8609628

[24] S. Zhu, M. Mac Kinnon, A. Carlos-Carlos, S. J. Davis, S. Samuelsen, Decarbonization will lead to more equitable air quality in California. Nat. Commun. **13**, 5738 (2022). 36180421 10.1038/s41467-022-33295-9 PMC9525584

[25] T. Carleton , Valuing the global mortality consequences of climate change accounting for adaptation costs and benefits*. Q. J. Eco. **137**, 2037–2105 (2022).

[26] R. K. Saari, Y. Mei, E. Monier, F. Garcia Menendez, Effect of health-related uncertainty and natural variability on health impacts and co-benefits of climate policy. Environ. Sci. Technol. **53**, 1098–1108 (2019). 30624913 10.1021/acs.est.8b05094

[27] E. J. Wilson, C. B. Christensen, S. G. Horowitz, J. J. Robertson, J. B. Maguire, “Energy Efficiency Potential in the U.S. Single-Family Housing Stock” (National Renewable Energy Lab. (NREL), Golden, CO (United States), (2017) (September 12, 2023), 10.2172/1414819.

[28] J. Lepeule, F. Laden, D. Dockery, J. Schwartz, Chronic exposure to fine particles and mortality: An extended follow-up of the harvard six cities study from 1974 to 2009. Environ. Health Perspect. **120**, 965–970 (2012). 22456598 10.1289/ehp.1104660 PMC3404667

[29] U.S. Environmental Protection Agency, “Guidelines for Preparing Economic Analyses” (2014) (April 25, 2023). Report EE-0568. https://www.epa.gov/environmental-economics/guidelines-preparing-economic-analyses.

[30] W. C. Baer, C. B. Williamson, The filtering of households and housing units. J. Plann. Literature **3**, 127–152 (1988).

[31] S. Ruggles , IPUMS USA: Version 12.0 [2020 COUNTYFIP RACE] (2022). 10.18128/D010.V12.0. Accessed 6 June 2023.

[32] S. M. Flood, L. C. Sayer, D. Backman, A. Chen, American Time Use Survey Data Extract Builder: Version 3.2 (2023) 10.18128/D060.V3.2 (March 10, 2023).

[33] S. Paltsev, E. Monier, J. Scott, A. Sokolov, J. Reilly, Integrated economic and climate projections for impact assessment. Clim. Change **131**, 21–33 (2015).

[34] J. D. Sacks , The Environmental Benefits Mapping and Analysis Program – Community Edition (BenMAP–CE): A tool to estimate the health and economic benefits of reducing air pollution. Environ. Modell. Soft. **104**, 118–129 (2018). PMC6022291 29962895

[35] J. B. Loomis, Updated Outdoor Recreation Use Values on National Forests and Other Public Lands (U.S. Department of Agriculture, Forest Service, Pacific Northwest Research Station, 2005).

[36] S. Ruggles , IPUMS USA: Version 12.0 [2020 COUNTYFIP PERSONAL INCOME] (2022) 10.18128/D010.V12.0 (June 6, 2023).

[37] H. Lee, J. Romero (eds.), “Climate Change 2023: Synthesis Report. Contribution of Working Groups I, II and III to the Sixth Assessment Report of the Intergovernmental Panel on Climate Change” (IPCC, 2023). 10.59327/IPCC/AR6-9789291691647.

[38] U. S. D. of L., Bureau of Labor Statistics, Civilian occupations required to spend the most time outdoors in 2020 at https://www.bls.gov/opub/ted/2021/civilian-occupations-required-to-spend-the-most-time-outdoors-in-2020.htm. The Economics Daily (2021).

[39] M. Henry, , “The 2020 Annual Homeless Report (AHAR) to Congress, Part 1: Point-in-Time Estimates of Homelessness” (U.S. Department of Housing and Urban Development, 2021) (March 31, 2023).

[40] J. D. East, E. Monier, F. Garcia-Menendez, Characterizing and quantifying uncertainty in projections of climate change impacts on air quality. Environ. Res. Lett. **17**, 094042 (2022).

[41] R. Tanzer-Gruener, J. Li, S. R. Eilenberg, A. L. Robinson, A. A. Presto, Impacts of modifiable factors on ambient air pollution: A case study of COVID-19 shutdowns. Environ. Sci. Technol. Lett. **7**, 554–559 (2020). 37566291 10.1021/acs.estlett.0c00365

[42] F. Yi, H. Ye, X. Wu, Y. Y. Zhang, F. Jiang, Self-aggravation effect of air pollution: Evidence from residential electricity consumption in China. Energy Econ. **86**, 104684 (2020).

[43] D. W. Abel , Air-quality-related health impacts from climate change and from adaptation of cooling demand for buildings in the eastern United States: An interdisciplinary modeling study. PLOS Med. **15**, e1002599 (2018). 29969461 10.1371/journal.pmed.1002599 PMC6029751

[44] H. Clarke , Health costs of wildfire smoke to rise under climate change. npj Clim Atmos Sci. **6**, 1–4 (2023).

[45] W. J. Fisk, W. R. Chan, Effectiveness and cost of reducing particle-related mortality with particle filtration. Indoor Air **27**, 909–920 (2017). 28170103 10.1111/ina.12371

[46] U.S. Energy Information Administration, Nearly 90% of U.S. households used air conditioning in 2020. Today in Energy (2022) (March 31, 2023).

[47] Y. He, V. Beck, Estimation of neutral plane position in high rise buildings. J. Fire Sci. **14**, 235–248 (1996).

[48] D. K. Farmer , Overview of HOMEChem: House observations of microbial and environmental chemistry. Environ. Science Process. Impacts **21**, 1280–1300 (2019). 10.1039/c9em00228f 31328749

[49] F. Henry, C. Coeur-Tourneur, F. Ledoux, A. Tomas, D. Menu, Secondary organic aerosol formation from the gas phase reaction of hydroxyl radicals with m-, o-and p-cresol. Atmos. Environ. **42**, 3035–3045 (2008).

[50] J. M. Logue, P. N. Price, M. H. Sherman, B. C. Singer, A method to estimate the chronic health impact of air pollutants in U.S. Residences. Environ. Health Perspect. **120**, 216–222 (2012). 22094717 10.1289/ehp.1104035 PMC3279453

[51] P. Azimi, B. Stephens, A framework for estimating the US mortality burden of fine particulate matter exposure attributable to indoor and outdoor microenvironments. J. Expo Sci. Environ. Epidemiol. **30**, 271–284 (2020). 30518794 10.1038/s41370-018-0103-4 PMC7039807

[52] R. J. Laumbach, K. R. Cromar, Personal interventions to reduce exposure to outdoor air pollution. Annu. Rev. Public Health **43**, 293–309 (2022). 34936825 10.1146/annurev-publhealth-052120-103607

[53] P. M. Reed , Multisector dynamics: Advancing the science of complex adaptive human-earth systems. Earth’s Future **10**, e2021EF002621 (2022).

[54] S. Elsawah , Eight grand challenges in socio-environmental systems modeling. Socio-Environ. Syst. Modell. **2**, 16226–16226 (2020).

[55] N. E. Selin, Lessons from a pandemic for systems-oriented sustainability research. Sci. Adv. **7**, eabd8988 (2021). 34039597 10.1126/sciadv.abd8988 PMC8153715

[56] B. Beckage , Linking models of human behaviour and climate alters projected climate change. Nat. Clim. Change **8**, 79–84 (2018).

[57] T. M. Bury, C. T. Bauch, M. Anand, Charting pathways to climate change mitigation in a coupled socio-climate model. PLoS Comput. Biol. **15**, e1007000 (2019). 31170149 10.1371/journal.pcbi.1007000 PMC6553685

[58] J. Menard, T. M. Bury, C. T. Bauch, M. Anand, When conflicts get heated, so does the planet: Coupled social-climate dynamics under inequality. Proc. R. Soc. B. **288**, 20211357 (2021). 10.1098/rspb.2021.1357 PMC8441127 34521252

[59] S. M. Constantino, M. Schlüter, E. U. Weber, N. Wijermans, Cognition and behavior in context: A framework and theories to explain natural resource use decisions in social-ecological systems. Sustain Sci. **16**, 1651–1671 (2021).

[60] I. Farahbakhsh, C. T. Bauch, M. Anand, Modelling coupled human–environment complexity for the future of the biosphere: Strengths, gaps and promising directions. Philos. Trans. R. Soc. B Biol. Sci. **377**, 20210382 (2022). 10.1098/rstb.2021.0382 PMC9234813 35757879

[61] J. Yoon , A typology for characterizing human action in multisector dynamics models. Earth’s Future **10**, e2021EF002641 (2022).

[62] B. D. Pienkosz, R. K. Saari, E. Monier, F. Garcia-Menendez, Natural variability in projections of climate change impacts on fine particulate matter pollution. Earth’s Future **7**, 762–770 (2019).

[63] A. Persily, A. Musser, S. J. Emmerich, Modeled infiltration rate distributions for U.S. housing. Indoor Air **20**, 473–485 (2010). 21070374 10.1111/j.1600-0668.2010.00669.x

[64] C. M. Long, H. H. Suh, P. J. Catalano, P. Koutrakis, Using time-and size-resolved particulate data to quantify indoor penetration and deposition behavior. Environ. Sci. Technol. **35**, 2089–2099 (2001). 11393992 10.1021/es001477d

[65] L. M. A. Bettencourt, Introduction to urban science: Evidence and Theory of Cities as Complex Systems (The MIT Press, 2021).

