# Supplementary material for: Health and equity implications of individual adaptation to air pollution in a changing climate
Source: Proc Natl Acad Sci U S A. 2024 Jan 16;121(5):e2215685121. doi: 10.1073/pnas.2215685121 (PMC10835109; doi:10.1073/pnas.2215685121)
Supplement: Supplementary file 1 — Appendix 01 (PDF) [file pnas.2215685121.sapp.pdf]

Supporting Information for
Health and Equity Implications of Individual Adaptation to Air Pollution in a Changing Climate.

Matt S. Sparks[a], Isaiah Farahbakhsh[b], Madhur Anand[b], Chris Bauch[c], Kathryn Conlon[d], James D. East[e,h], Tianyuan Li[a], Megan Lickley[f,i,j], Fernando Garcia-Menendez[e], Erwan Monier[g], Rebecca K. Saari[a]

[a] Department of Civil and Environmental Engineering, University of Waterloo, ON, CA N2L 3G1

[b] School of Environmental Sciences, University of Guelph, Waterloo, ON, CA N1G 2W1

[c] Department of Applied Mathematics, University of Waterloo, ON, CA N2L 3G

[d] School of Medicine Department of Public Health Sciences and School of Veterinary Medicine Department of Medicine and Epidemiology, University of California, Davis, Davis, CA, USA, 95616

[e] Department of Civil, Construction and Environmental Engineering, North Carolina State University, Raleigh, NC, USA, 27695

[f] Department of Earth, Atmospheric and Planetary Sciences, Massachusetts Institute of Technology, Cambridge, MA, USA, 02139N

[g] Department of Land, Air and Water Resources, University of California, Davis, Davis, CA, USA, 95616

[h] Current Affiliation: School of Engineering and Applied Sciences, Harvard University, Cambridge, MA, 02138

[i] Current Primary Affiliation: The Earth Commons—Georgetown University's Institute for Environment & Sustainability, Washington, DC, 20057

[j] Current Secondary Affiliation: Science, Technology, and International Affairs, School of Foreign Service, Georgetown University, Washington, DC, 20057

*Rebecca K. Saari

Email: rsaari@uwaterloo.ca

This PDF file includes:

Supporting text
Figures S1 to S2
Tables S1 to S15
SI References

SUPPLEMENTAL METHODS

$PM_{2.5}$ Infiltration. The infiltration factor F_{INF} has been shown to be variable in previous studies. A national study by Persily et al. (2010) estimates that leakage accounts for a varying amount of infiltration based on the age and type of the dwelling unit (1). Using 2015 U.S. Census American Community Survey data on housing age and the proportion of population living in single-family housing, we calculated the weighted average of ACH due to leakage across the country as approximately 0.33 (2). Based on the plots by Long et al. (2001), an ACH rate of 0.33 corresponds with a $PM_{0.7\text{-}2.5}$ infiltration rate of approximately 20% (3). We set F_{INF} = 0.2. Outdoor $PM_{2.5}$ concentrations vary by climate policy, day, and grid cell, and are updated based on model outputs.

Time Use. We use an average daily time spent outdoors of one hour. To determine T_{out}, we reviewed the ATUS 2021 Activity Summary File, analyzing the data of each respondent, and the location of their activity data according to the classification system developed by Hoehne et al. (2018) (4). In their study, Hoehne et al. (2018) classified activities as occurring "Indoor", "Outdoor", or "Unknown" (4). It is important to note that outdoor work is not accounted for in that study, leading to an underestimate of total outdoor time.

By applying these classifications to the ATUS 2021 data, we found that T_{out} – meaning activities classified as "Outdoor" – varied among respondents from 0 – 15.5 hours. The mean T_{out} value was 34 minutes per day. The mean time spent in "Unknown" locations was 45 minutes per day. This leads to a range of 34 – 79 minutes per day before accounting for outdoor work. The study by Cox-Ganser and Henneberger (2021) reported that 14.5 million people worked jobs classified as outdoors in 2019 (5). Based on the 2019 U.S. population of 328.3 million, this represents about 4.4% of the population. If we estimate that these people work 8 hours per day on 5 out of 7 days per week, this leads to an increase in the population-weighted average time spent outside of about 15 minutes per day, which brings the range of average time outside to 49 – 94 minutes per day.

To reduce the risk of being influenced too strongly by the COVID-19 pandemic, we also examined the ATUS 2019 Activity Summary File data (6). That data showed a range of daily time spent outdoors of 0 – 17 hours. The mean time outdoors value was 29 minutes per day and the mean unknown time was 52 minutes per day. After accounting for the population who works outdoors, the range of average time spent outside would be between 44 – 96 minutes a day.

Social Learning Model

Overview. Our social learning model represents the spread of adaptation behavior through a population through comparison of the utility between adapters and non-adapters. In this model, we define separate regions, *i,* which represent one 2 x 2.5-degree grid cell. Each region has a homogenous population of fixed size at each time step. Individuals in this population can either be adapters or non-adapters. We define the proportion of adapters as x_i, thus $1 - x_i$ is the proportion of non-adapters. In this population, social norms encourage the dominant behavior. The utilities of being adapters and non-adapters are:

$$U_{A,i} = -C_i + \delta_r \omega(x_i) \tag{S1}$$

$$U_{N,i} = -R_i + \delta_r (1 - \omega(x_i)) \tag{S2}$$

In these equations, C_i represents the cost associated with adaptation in region i. R_i is a function that represents the perceived risk of negative health effects due to air pollution in region i. An adapter believes they mitigate their risk of these negative health effects by adapting, whereas a non-adapter receives the full amount of this perceived health risk. δ_r represents the weight of social norms in each U.S census region and $\omega(x_i)$ is a function that determines the social norm that is predominant for grid cell i.

We subtract $U_{A,i} - U_{N,i}$ to define ΔU_i as:

$$\Delta U_{i,t} = -C_{i,t} + R_{i,t} + \delta_r (2\omega(x_{i,t}) - 1) \tag{S3}$$

Where the parameters remain as defined in equations S1 and S2. Since C_i and R_i are measured in dollars and take on large values of magnitude much greater than 1, they will be scaled to be unitless and close to the range of [-1, 1], which is a similar order of magnitude for the social norms term. We do so by dividing the difference between $R_{i,t}$ and $C_{i,t}$ by an empirically fitted constant, R_0.

$$\Delta U_{i,t} = \frac{R_{i,t} - C_{i,t}}{R_0} + \delta_r(2\omega(x_{i,t}) - 1) \tag{S4}$$

Where R_0 is an empirically fitted constant added to scale the difference between mitigated perceived risk and cost such that its influence is of similar magnitude to the influence of social norms.

In each grid cell, individuals will update their adaptation/non-adaptation decision based on the rate at which information is spread throughout their social networks, the proportion of the population who are currently adapters or non-adapters, and the difference in utility between the two strategies as calculated in equation S4. The proportion of adapters in grid cell i at time t+1 is governed by the discrete social learning model (equation S5) where one time step is equal to one quarter, or 3-month period.

$$\tilde{x_{i,t+1}} = x_{i,t} + \kappa_r \sigma_i x_{i,t}(1 - x_{i,t})\Delta U_{i,t} \tag{S5}$$

Where $x_{i,t}$ is the proportion of adapters in grid cell i at time t, $\Delta U_{i,t}$ is the difference in utility between adaptation and non-adaptation $(U_{A,i} - U_{N,i})$. κ_r denotes the social learning rate in each U.S. census region, and σ_i is the population of gridcell i. Population is included because higher population areas lead to more diverse social networks for inhabitants which increases the spread of information (7). The proportion of adapters in a grid cell must be between 0 (no adapters in the population) and 1 (the entire population is an adapter). Equation S6 enforces that bound.

$$x_{i,t+1} = max(min(\tilde{x_{i,t+1}}, 1), 0) \tag{S6}$$

Determining Social Influence. In this model, we include the spread of information and influence across grid cells. We do this by defining $\omega(x_i)$, the social behavioral norm for grid cell i, as:

$$\omega(x_{i,t}) = \sum_j SCI_{ij} x_{j,t} \tag{S7}$$

Where $SCI_{i,j}$ is the normalized Social Connectedness Index (SCI) between two grid cells. SCI is defined by Meta/Facebook as the relative likelihood of a friendship link between users in two locations (8). We use this as a proxy for information spread between two locations, which influences social norms. To calculate $SCI_{i,j}$, we retrieved SCI values at the U.S. county-county level from Facebook. Then, we aggregated the county-county connections to the grid cell – grid cell level using the process introduced in (9). The process begins by calculating the portion of counties that are in each grid cell. We then weight each county by its proportion of the total grid cell population. For each grid cell pair, we multiply $county_i$ – $county_j$ SCI values by the proportion of population that $county_i$ and $county_j$ make up. We sum these values to get the SCI between grid cells. Then, we multiply the SCI value between two grid cells by the population of both grid cells to get a relative number of connections between the grid cells. We then calculate the sum of the connections each grid cell has and normalize each grid cell connection total to determine the relative influence that each cell has on each other. The social norm for a grid cell is then calculated as the product of relative influence from grid cell to grid cell (SCI_{ij}) and the proportion of adapters in each influencing grid cell (x_j).

Calculating Marginal Costs. Marginal costs within the Social Learning Model are calculated similarly to the Rational Actor Model. As most literature surveys show that curtailing outdoor activities is the predominant adaptation strategy in the general population, we set the cost of adaptation to be proportional to the reduction of time spent outdoors by adapters. We value lost time outdoors at the average wage rate. The cost of adaptation can be represented as:

$$C_{i,t} = \varphi_i \times T_{adapt} \quad \text{(S8)}$$

$$C_{i,t} = \varphi_i \times \rho_{i,t} \times \xi_i \times T_{out} \quad \text{(S9)}$$

Where φ_i is the mean hourly wage rate in grid cell i. ξ_i is the proportion of an air quality alert day that an adapter chooses to spend indoors and is empirically fit by U.S. census region. ρ_i is the proportion of $PM_{2.5}$ – driven air quality alert days within grid cell i over the current time period. T_{out} is the national average time spent outdoors, which is 2 hours per day over 365 days per year.

Calculating Perceived Risk. The perceived risk function $R_{i,t}$ includes a term for likelihood of risk, which is a function of the proportion of bad air days in the current time step, $\rho_{i,t}$, a term for severity of risk, $M_{i,t}$, and a scaling factor s.

$$R_{i,t} = s \times \rho_{i,t} \times M_{i,t} \quad \text{(S10)}$$

The term for the severity of risk is based on $M_{\tilde{i}}$, which is defined as the annual per-capita costed premature mortality burden due to $PM_{2.5}$, which is updated at quarterly intervals.

$$M_{i,t} = VSL(1 - \left(1 - \eta_{i,t}\right) \times x_{i,t})M_{\tilde{i,t}} \quad \text{(S11)}$$

The costed premature mortality burden experienced by a grid cell of adapters and non-adapters is dependent on several factors. First is c, the Value of a Statistical Life, which is based on a population's willingness to pay to reduce their risk of premature death. Second is $\eta_{i,t}$, which is the proportion by which adapters reduce their $PM_{2.5}$ exposure across the time period by adapting. Third is $x_{i,t}$, the proportion of the population who are adapters in the current time period. Last is $M_{\tilde{i,t}}$, which is the incidence of PM-driven premature mortality for a population of non-adapters.

The adaptation strategy for adapters is to reduce their time outdoors on days with an AQI > 100. The proportion of days with AQI > 100 also termed "Air Quality Alert Days" in each grid cell/time period is $\rho_{i,t}$. AQI > 100 is the threshold for "Unhealthy for Sensitive Groups" and is the point where the U.S. EPA begins recommending behavior change. When projected AQI exceeds this threshold, health ministries may generate an alert to people living nearby. Therefore, the term $\eta_{i,t}$ is calculated as:

$$\eta_{i,t} = \frac{\sum_{d=1}^{\rho_{i,t}\times 365} \psi \times PM[PM > 35.4](d) + \sum_{d=1}^{365 - \rho_{i,t}\times 365} PM[PM <= 35.4](d)}{\sum_{d=1}^{365} PM(d)} \quad \text{(S12)}$$

Where, similarly to the Rational Actor Model, the term ψ is the ratio of the exposure an adapter would experience when staying indoors on an alert day to the exposure one has when they do not stay indoors. This term is dependent on ξ_i and F_{INF} and is defined as:

$$\psi_i = \frac{\frac{T_{in,day} + \xi_i T_{out,day}}{24} \times F_{INF} + \frac{(1-\xi_i)T_{out,day}}{24}}{\frac{T_{in,day}}{24} \times F_{INF} + \frac{T_{out,day}}{24}} \quad \text{(S13)}$$

PM is the set of daily 24-hour mean $PM_{2.5}$ concentrations over a given year. The set is separated into two subsets, one that includes the $PM_{2.5}$ concentrations above 35.4 $\frac{\mu g}{m^3}$ and one that is below or equal to 35.4 $\frac{\mu g}{m^3}$. This distinction is made per the concentration values in Table S1 as a $PM_{2.5}$ concentration of 35.4 $\frac{\mu g}{m^3}$ corresponds with an AQI value of 100.

Fitting the Behavior Model. Fitting the parameters κ_r, δ_r, ξ_r, and R_0 was done by minimizing error between the census-region aggregated Social Learning model and annual empirical values of adapters from the SummerStyles datasets. Equations S14-S16 were used to fit the behavioral model:

$$X_{r,t} = \sum_i p_i \mu_{i,r} x_{i,t} \quad \text{(S14)}$$

$$X_{r,t} = \sum_i p_i \mu_{i,r} (max(min(x_{i,t-1} + \kappa_r x_{i,t-1}(1 - x_{i,t-1}) \Delta U_{i,t-1}(\xi_r \delta_r R_0),1),0) \quad \text{(S15)}$$

$$\Delta U_i = \frac{R_i(\xi_r) - \beta_i(\xi_r)}{R_0} + \delta_r(2\omega(x_i) - 1) \quad \text{(S16)}$$

Where r is one of the four U.S. census regions and $\mu_{i,r}$ represents the proportion of cell i in region r. We use a national, population-weighted hourly wage rate, ϕ, as we found that fitting using average wage by grid cell led to unrealistic results that were highly sensitive to grid-level wage values. Low-wage grid cells tended to equilibrate to full adaptation and high-wage grid cells tended to equilibrate to pure non-adapters within the fitting period of 2014-2020. Since adapters are updated quarterly and our data on adapters is yearly, X_r is interpolated to quarterly data. Also, throughout the fitting process, $X_{r,0}$ are fit and allowed to vary by 25% of their empirical value.

SUPPLEMENTAL RESULTS

Projection Into Future. For running the model in the years 2050 and 2100, the proportion of adapters is initialized to the last values from the previous time period. For 2050, the proportion of adapters from 2020 taken from the historical fitting process is used. Additionally, population and economic values are adjusted for predicted future growth, which are period and sometimes policy dependent and summarized in Tables S6 though S9.

Comparison to Start-of-Century. Table S10 compares $PM_{2.5}$ exposure, adaptation days, exposure reduction per adaptation, adaptation benefits, adaptation costs, and adaptation net benefits between different policy/time period combinations and the beginning of the 21st century. Table S10 shows that in both the REF and P3.7 scenarios, exposure to $PM_{2.5}$ without adaptation will increase by the year 2050. P3.7 reduces the climate penalty on $PM_{2.5}$ pollution by approximately half at 2050 and maintains pollution levels throughout the remainder of the century. Under REF, $PM_{2.5}$ exposures continue to rise, leading to a climate penalty that is over five times worse than P3.7. Rational adaptation is shown to mitigate $PM_{2.5}$ exposure in the future. At 2050, rational adapters would, on average, adapt 27 more days per year than they would under REF and 25 more days per year than they would under P3.7. This increase in adaptation is caused by higher $PM_{2.5}$ pollution concentrations and an older population who will be more strongly affected by $PM_{2.5}$ pollution. We see that these populations are willing to adapt when pollution reductions per adaptation day are lower than they would be at the start-of-century. The adaptation benefits, costs, and net benefits show that adaptation net benefits are highest in the REF scenario when pollution levels are highest and decrease under stringent climate policy.

Quantifying the Comparison to Previous Research. The most similar previous research to this is Buonocore et al. (2021), who investigated the potential health benefits of air quality alerts **(10)**. Buonocore et al. (2021) consider both long- and short-term mortality risk changes due to ozone and $PM_{2.5}$ in the population aged 65 years and older. When the Air Quality Index exceeds 100, assuming no infiltration or offsetting indoor sources, they find per capita benefits to be no higher than $14 per person per hour. Here, we find an average benefit of $31 per person per hour for adults aged 30 and over at the start-of-century across the U.S. Several differences in study design help to explain this difference; however, the dominant factor is the treatment of exposure reduction. Buonocore et al. allows a single hour of adaptation per day between 6 am and 9 pm, with exposure to outdoor air at all other hours of the day. This leads to differing exposure reductions based on how much worse the most polluted hour is than the other 23 hours. Our adaptation model, due to using 24-hour mean concentrations and accounting for exposure differences indoors and outdoors, results in a constant exposure reduction of approximately 15%. In Buonocore et al. (2021), to achieve that level of reduction, the worst hour would have to be four times higher than the average of the remaining 23 hours. When we reproduce Buonocore et al.'s findings but with our exposure reduction assumptions, we find up to a 1.5-fold (or 50%) increase in benefits.

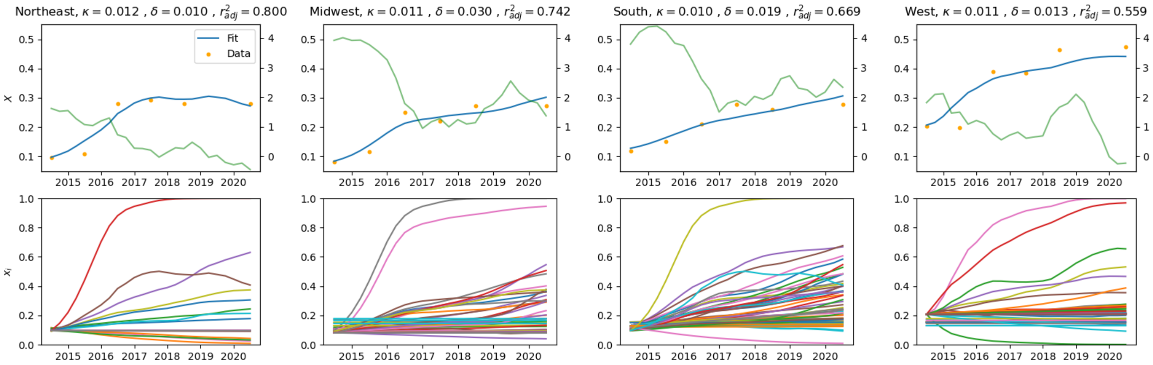

Figure S1. Best fit for Social Learning Model parameters based on fitting to SummerStyles survey data by region **(11)**. Top row of plots show the national proportion of adapters based on survey data along with the modeled best fit and difference in utility between adapters and non-adapters. The bottom row of plots show the proportion of adapters by grid cell over the fitting period (2014-2020).

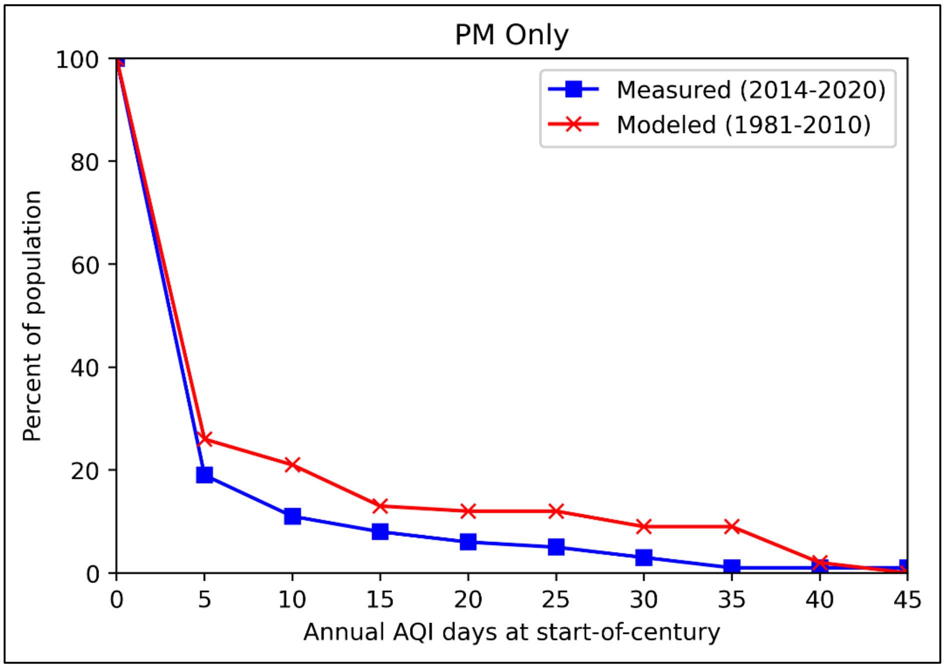

Figure S2. Comparison of modeled AQI days vs measured AQI days. Measured AQI days based on $PM_{2.5}$ concentrations from US EPA monitors interpolated with Voronoi neighbor averaging performed in BenMAP-CE.

.

Table S1. U.S. EPA guidance for PM-driven AQI. Adapted from the "Technical Assistance Document for the Reporting of Daily Air Quality – the Air Quality Index (AQI)" document (1).

**AQI Range**	**Category**	**$PM_{2.5}$ Range (µg/m3)**	**U.S. EPA Guidance**
**0 - 50**	Good	0 – 12.0	None
**51 – 100**	Moderate	12.1 – 35.4	Unusually sensitive people should consider reducing prolonged or heavy exertion.
**101 – 150**	Unhealthy for Sensitive Groups	35.5 – 55.4	People with heart or lung disease, older adults, children, and people of lower socioeconomic status should reduce prolonged or heavy exertion.
**151 – 200**	Unhealthy	55.5 – 150.4	People with heart or lung disease, older adults, children, and people of lower socioeconomic status should avoid prolonged or heavy exertion; everyone should reduce prolonged or heavy exertion.
**201 – 300**	Very Unhealthy	150.5 – 250.4	People with heart or lung disease, older adults, children, and people of lower socioeconomic status should avoid all physical activity outdoors. Everyone else should avoid prolonged or heavy exertion.
**301 – 500**	Hazardous	250.5 – 500.4	Everyone should avoid all physical activity outdoors; people with heart or lung disease, older adults, children, and people of lower socioeconomic status should remain indoors and keep activity levels low.

Table S2. Parameter definitions for the Rational Actor Model

Name	Description
$\boldsymbol{NB_d}$	Net benefits of adapting on day d.
$\boldsymbol{B_d}$	Benefit of adapting on day d.
$\boldsymbol{C_d}$	Cost of adapting on day d.
$\boldsymbol{VSL}$	Value of a Statistical Life. Calculated from the willingness to pay to reduce risk of death, with risk reduction normalized to 1.
$\boldsymbol{I_d}$	Change in annual mortality incidence rate due to adaptation on a given day.
$\boldsymbol{Y_0}$	Population baseline mortality rate.
$\boldsymbol{RR}$	Relative Risk. The increase in incidence rate of an adverse health outcome (mortality in this study) that can be attributed to a $10\frac{\mu g}{m^3}$ rise in $PM_{2.5}$ concentration.
$\boldsymbol{\Delta PM_{2.5,a_d}}$	Change in annual 24-hour mean $PM_{2.5}$ concentration after adaptation on day d.
$\boldsymbol{PM_{2.5,a}}$	Average annual outdoor concentration of $PM_{2.5}$ with no adaptation.
$\boldsymbol{PM'_{2.5,a_d}}$	Equivalent annual outdoor concentration of $PM_{2.5}$ after adaptation on day d.
$\boldsymbol{PM_{2.5,n}}$	24-hour mean outdoor $PM_{2.5}$ concentration on each non-adaptation day n.
$\boldsymbol{PM_{2.5,d}}$	24-hour mean outdoor $PM_{2.5}$ concentration on each adaptation day d.
$\boldsymbol{\psi}$	Adaptation exposure reduction factor.
$\boldsymbol{T_{in}}$	Average daily time spent indoors. 23 hours for default case.
$\boldsymbol{T_{out}}$	Average daily time spent outdoors. 1 hour for default case.
$\boldsymbol{F_{INF}}$	Infiltration Factor. Represents the fraction of outdoor $PM_{2.5}$ that enters indoors and remains airborne.
$\boldsymbol{\varphi}$	Hourly adaptation cost. Default parameterization for a grid cell is that grid cell's mean hourly wage.
$\boldsymbol{T_{adapt}}$	Time spent adapting in hours. For Rational Actor model, this is set equal to $\boldsymbol{T_{out}}$ to maximize net benefits of adapting.

Table S3. Parameter definitions for Social Learning Model.

Name	Description
$\boldsymbol{x_i}$	Proportion of adapters in grid cell i.
$\boldsymbol{1 - x_i}$	Proportion of non-adapters in grid cell i.
$\boldsymbol{U_{A,i}}$	Utility of being an adapter.
$\boldsymbol{\beta_i}$	Cost of adaptation.
$\boldsymbol{\delta_r}$	Weight of social norms in U.S. census region r.
$\boldsymbol{\omega(x_i)}$	Function to determine social norms.
$\boldsymbol{U_{N,i}}$	Utility of being a non-adapter.
$\boldsymbol{R_i}$	Function that represents the portion of perceived risks of air pollution that are mitigated by adapting.
$\boldsymbol{\Delta U_{i,t}}$	The difference in utility between being an adapter and a non-adapter.
$\boldsymbol{R_0}$	Empirically fitted constant added to scale the difference between mitigated perceived risk and cost to a comparable unit to the influence of social norms.
$\boldsymbol{\widetilde{x_{i,t+1}}}$	Raw proportion of adapters in grid cell i one time step into the future. Gets bound by minimum and maximum values.
$\boldsymbol{x_{i,t}}$	Proportion of adapters in grid cell i in the current time step.
$\boldsymbol{\kappa_r}$	Social learning rate for U.S. census region r.
$\boldsymbol{\sigma_i}$	Parameter related to the population in grid cell i. Higher values mean more population.
$\boldsymbol{SCI_{ij}}$	Social Connectedness Index between grid cells i and j. Determines weight of social norm influence between the two grid cells.
$\boldsymbol{\beta_{i,t}}$	Cost of adaptation in grid cell i at time t.
$\boldsymbol{\varphi_i}$	Mean hourly wage in gird cell i.
$\boldsymbol{T_{adapt}}$	Daily time that the population spends adapting.
$\boldsymbol{\xi_r}$	The portion of T_{out} that the population switches to T_{in} when adapting.
$\boldsymbol{\rho_{i,t}}$	The proportion of days with AQI over the relevant threshold (100 or 150) in a grid cell i at time t.
$\boldsymbol{s}$	An empirically fit scaling factor relating perceived risk to actual risk.
$\boldsymbol{M_{i,t}}$	Represents the economic value of the severity of risk of premature death due to $PM_{2.5}$ pollution in grid cell i at time t.
$\boldsymbol{\eta_{i,t}}$	The proportion by which adapters reduce their $PM_{2.5}$ exposure in grid cell i and time t.
$\boldsymbol{VSL}$	The Value of a Statistical Life. Determined by a population's willingness to reduce their risk of premature death.
$\boldsymbol{\widetilde{M_{i,t}}}$	The incidence of $PM_{2.5}$ driven premature mortality for a population of non-adapters.
$\boldsymbol{\psi}$	Ratio of exposure under adaptation to exposure under non-adaptation time use. $\left({Exp_{day,adapt}}/{Exp_{day,base}} \right)$
$\boldsymbol{F_{INF}}$	Fraction representing the portion of outdoor $PM_{2.5}$ that penetrates the building envelope and remains suspended indoors.

Table S4. Portion of health burden valuation driven by $PM_{2.5}$ mortality. Calculated by isolating the valued health burden from $PM_{2.5}$ mortality and dividing that by the total health burden from $PM_{2.5}$ and O_3. Data for calculations are in Saari et al. (2019) (7).

Year	P3.7
**2050**	94.68%
**2100**	93.20%

Table S5. Parameters used for Social Learning Model. These parameters (except for T_{out}, which was determined based on previous studies) are empirically fit through the process described above.

Name	Description	Value
**s**	Scaling factor for risk	1
R_0	Baseline risk perception ($ USD 2000)	446
ξ_n	Proportion of outdoor time adapters choose to stay indoors in Northeast	0.7949
ξ_m	Proportion of outdoor time adapters choose to stay indoors in Midwest	0.7749
ξ_s	Proportion of outdoor time adapters choose to stay indoors in South	0.8492
ξ_w	Proportion of outdoor time adapters choose to stay indoors in West	0.7047
T_{out}	Average time spent outdoors for non-adapters (hours/day)	1
κ_n	Social learning rate for Northeast census region	0.01205
κ_m	Social learning rate for Midwest census region	0.01160
κ_s	Social learning rate for South census region	0.01001
κ_w	Social learning rate for West census region	0.01172
δ_n	Strength of social norms for Northeast census region	0.01095
δ_m	Strength of social norms for Midwest census region	0.03052
δ_s	Strength of social norms for South census region	0.01900
δ_w	Strength of social norms for West census region	0.01313

Table S6. Climate mitigation policy scenarios included in this study.

Year	Mitigation Policy
2050	REF
	3.7 W/m^2
2100	REF
	3.7 W/m^2

Table S7. Population projection factors and data sources.

Category	Description	Year	Value	Source
Population Projection	Population Ratio of Year vs. Base Year of 2005	2050	1.48	Population growth based on long-term trends of United Nations Data, with details provided in Paltsev et al. (2015)
		2100	1.73	

Table S8. Economic projection factors (GDP per capita). Data based on results from Paltsev et al. (2015).

	**Scenario**	
**Year**	**REF**	**POL3.7**
**2050**	2.01	1.82
**2100**	3.94	3.37

Table S9. Baseline mortality projection factors based on Paltsev et al. (2015).

Category	Description	Year	Value	Source
**Mortality Projection**	Baseline Mortality Ratio of Year vs. Base Year of 2008 (Ozone)	2050	1.24	Respiratory Mortality Incidence Rates from International Futures as provided in West et al. (2013)
		2100	1.64	
	Baseline Mortality Ratio of Year vs. Base Year of 2008 ($PM_{2.5}$)	2050	1.12	Cardiovascular Mortality Incidence Rates from International Futures as provided in West et al. (2013)
		2100	0.92	

Table S10. Changes in population weighted national mean attributes compared to start-of-century. The values in this table are the difference in national population weighted exposure data and adaptation benefits and costs between future time periods and the reference period for the year 2000.

Scenario	Exposure without Adaptation (μg/m^3)	Exposure under Rational Adaptation (μg/m^3)	Adaptation Days (Days/Year)	Exposure Reduction per Adaptation Day (μg/m^3)	Adaptation Benefits (Trillion USD 2020)	Adaptation Costs (Trillion USD 2020)	Adaptation Net Benefits (Trillion USD 2020)
**REF 2050**	0.554	-0.062	27	-0.003	0.516	0.330	0.186
**P37 2050**	0.278	-0.164	25	-0.004	0.472	0.307	0.166
**REF 2100**	1.472	0.416	19	0.001	0.968	0.609	0.359
**P37 2100**	0.260	-0.053	12	-0.001	0.651	0.426	0.225

Table S11. Effect of optimal adaptation on health effects of policy. The "Mitigate" scenarios are the costs and benefits accrued by enacting climate change mitigation policy and not considering $PM_{2.5}$ adaptation. The "Rational Adapt" cases look at how including rational adaptation in the analysis reduces the cost (by reducing adaptation days from REF to P3.7) and reduces the benefits (because adaptation reduces the $PM_{2.5}$ health burden). Policy benefits are reduced more than policy costs, leading to smaller benefit / cost ratios.

**Case**	**Costs (Trillion USD 2020)**	**Costs Per Capita (USD 2020)**	**Benefits in POL Econ (Trillion USD 2020)**	**Benefits Per Capita (USD 2020)**	**% of costs offset (B/C ratio)**	**% change from Base of B/C ratio**
**Mitigate POL 3.7 2050**	5.042	18,800	0.295	1,100	6%	N/A
**Rational Adapt POL 3.7 2050**	5.019	18,700	0.252	940	5%	-14%
**Mitigate POL 3.7 2100**	17.042	54,400	2.304	7,400	14%	N/A
**Rational Adapt POL 3.7 2100**	16.860	53,900	1.987	6,300	12%	-13%

Table S12: $PM_{2.5}$-driven AQI alert days per year if threshold for alerts is > 150 compared to the number of rational adaptation days.

Scenario	Mean AQI Alert Days (Min, Max)	Rational Adaptation Days (Min, Max)
**REF 2000**	1 (0, 2)	7 (6, 9)
**REF 2050**	5 (4, 6)	34 (30, 37)
**P37 2050**	4 (3, 6)	32 (29, 35)
**REF 2100**	7 (5, 9)	26 (22, 30)
**P37 2100**	4 (3, 6)	19 (16, 21)

SI References

1. A. Persily, A. Musser, S. J. Emmerich, Modeled infiltration rate distributions for U.S. housing. *Indoor Air* **20**, 473–485 (2010).

2. U.S. Census Bureau, 2015 American Community Suvey (2015).

3. C. M. Long, H. H. Suh, P. J. Catalano, P. Koutrakis, Using time-and size-resolved particulate data to quantify indoor penetration and deposition behavior. *Environmental science & technology* **35**, 2089–2099 (2001).

4. C. G. Hoehne, *et al.*, Heat exposure during outdoor activities in the US varies significantly by city, demography, and activity. *Health & Place* **54**, 1–10 (2018).

5. J. M. Cox-Ganser, P. K. Henneberger, Occupations by Proximity and Indoor/Outdoor Work: Relevance to COVID-19 in All Workers and Black/Hispanic Workers. *Am J Prev Med* **60**, 621–628 (2021).

6. U.S. Bureau of Labor Statistics, American Time Use Survey 2019 (2019).

7. L. M. A. Bettencourt, *Introduction to urban science: evidence and theory of cities as complex systems* (The MIT Press, 2021).

8. M. Bailey, R. Cao, T. Kuchler, J. Stroebel, A. Wong, Social Connectedness: Measurement, Determinants, and Effects. *Journal of Economic Perspectives* **32**, 259–280 (2018).

9. M. Bailey, *et al.*, International trade and social connectedness. *Journal of International Economics* **129**, 103418 (2021).

10. J. J. Buonocore, L. A. Robinson, J. K. Hammitt, L. O'Keeffe, Estimating the Potential Health Benefits of Air Quality Warnings. *Risk Analysis* **41**, 645–660 (2021).

11. M. C. Mirabelli, S. Ebelt, S. A. Damon, Air Quality Index and air quality awareness among adults in the United States. *Environmental Research* **183** (2020).

12. S. Paltsev, E. Monier, J. Scott, A. Sokolov, J. Reilly, Integrated economic and climate projections for impact assessment. *Climatic Change* **131**, 21–33 (2015).
